# The Role of Morphine-Induced Impairment of Intestinal Epithelial Antibacterial Activity in Dysbiosis and its Impact on the Microbiota-Gut-Brain Axis

**DOI:** 10.21203/rs.3.rs-3084467/v1

**Published:** 2023-07-18

**Authors:** Hamid Akbarali, Karan Muchhala, Minho Kang, Eda Koseli, Justin Poklis, Qingguo Xu, William Dewey, Jennifer Fettweis, Nicole Jimenez

**Affiliations:** Virginia Commonwealth University; Virginia Commonwealth University; Virginia Commonwealth University; Virginia Commonwealth University; Virginia Commonwealth University; Virginia Commonwealth University; Virginia Commonwealth University; Virginia Commonwealth University; Virginia Commonwealth University

## Abstract

Host-produced antimicrobial peptides (AMPs) are critical for the integrity of the gut epithelial barrier as they prevent the pathogenesis of the enteric microbiota. Here, we report that intestinal antimicrobial activity is reduced, and epithelial permeability is increased in a morphine-dependent mouse model. Antimicrobial activity and permeability are restored by fecal transplant (FMT) from morphine-naïve mice or by oral gavage of sodium butyrate. Butyrate levels are reduced in the fecal samples of morphine-treated mice concomitant with a reduction in the phylum, *Firmicutes*. The alpha diversity of the microbiome is also restored by oral butyrate in morphine-dependent mice. FMT or sodium butyrate prevents downregulation of the antimicrobial peptide, Regenerating islet-derived 3 gamma (Reg3γ), and the development of antinociceptive tolerance in morphine-dependent mice. These data implicate impairment of the antimicrobial activity of the intestinal epithelium as a mechanism by which morphine disrupts the microbiota-gut-brain axis.

## INTRODUCTION

It is well established that the chronic use of μ-opioid analgesics such as morphine results in the development of tolerance to the antinociceptive effects. Thus, a larger dose may be required for adequate pain control. Dose escalation can increase the propensity for unwanted effects such as addiction, constipation, and death due to overdose in severe cases since tolerance to opioid-induced respiratory depression does not develop at the same rate as tolerance to antinociception ^1^. Despite the known risks, opioids remain the gold standard for managing pain in the clinic. It is, therefore, important to 1) understand the mechanisms that form the basis for the development of tolerance and 2) apply this information to formulate novel strategies to confront the ongoing opioid crisis.

Traditionally, nausea, vomiting, and constipation are reported as common gastrointestinal effects of μ-opioid analgesics^2^. Recent clinical evidence indicates that opioid use is associated with intestinal dysbiosis^3–7^. While nutritional deficiencies, poor hygiene, and comorbidities can contribute to an altered microbiota in opioid users^8–10^, the absence of a microbial shift in opioid users treated with opioid receptor antagonists, naloxone or naltrexone^4^, suggests that the dysbiosis may be an opioid receptor-dependent effect.

In addition to the burgeoning clinical evidence for opioid-induced dysbiosis, several preclinical studies have reported changes in the intestinal microbiome with chronic opioid exposure. Mice chronically treated with opioids exhibit altered intestinal microbiota, intestinal inflammation, perturbed intestinal epithelial barrier, and systemic translocation of luminal bacteria^11–18^. The exact sequence of events is unclear. Several studies have demonstrated that the disruptive effects of opioids on the intestinal microbiota and the intestinal epithelium contribute to the development of tolerance to opioid-induced antinociception *in vivo*^11,12,16^ and in primary afferent dorsal root ganglia neurons^11,12^ as noted by the reversal of *in vivo* and cellular tolerance to morphine after antibiotic treatment. However, there is a significant gap in our current understanding of how opioids induce dysbiosis and its impact on antinociceptive tolerance.

The host and autochthonous microbiota maintain a commensal relationship through several mechanisms, including the secretion of antimicrobial proteins and peptides (AMPs) and IgA^19,20^. AMPs are released primarily by specialized epithelial cells called Paneth cells that reside within small intestinal crypts^20^. Enterocytes in the villi also secrete AMPs^21^. AMPs help create a chemical barrier against bacterial colonization of the intestinal epithelium and maintain the composition and diversity of the intestinal microbiota by inhibiting pathogenic strains. Indeed, several studies have demonstrated that depletion of Paneth cells, or disruption of mechanisms that regulate the synthesis or secretion of AMPs, enhances susceptibility to intestinal inflammation, promotes systemic translocation of luminal bacteria, and increases vulnerability to pathogenic infections^20^. Since chronic treatment with morphine alters the intestinal microbiome and results in bacterial translocation, we sought to examine whether morphine impairs antimicrobial activity of the *intestinal epithelium*.

Interestingly, fecal samples of opioid users were deficient in the short-chain fatty acid (SCFA), butyrate, and butyrate-producing bacteria^4,7^. Anerobic bacteria in the intestines produce butyrate by fermenting dietary fiber and resistant starches^22^. Butyrate is utilized as a significant energy source by colonocytes; it improves epithelial barrier function by inducing the expression of tight junction proteins, increasing AMP production, and inhibiting pro-inflammatory cytokines^22^. We previously reported that exacerbating intestinal inflammation in an experimental model of colitis increased the rate at which tolerance developed to morphine’s antinociceptive effects^18^. Since butyrate is critical for maintaining the physiology of the intestinal mucosa and inhibits intestinal inflammation, in the present study, we tested the effect of butyrate on epithelial barrier function, and on the development of tolerance to morphine-induced antinociception.

## RESULTS

### Chronic morphine exposure alters the composition of the fecal microbiota.

**Figure 1:**
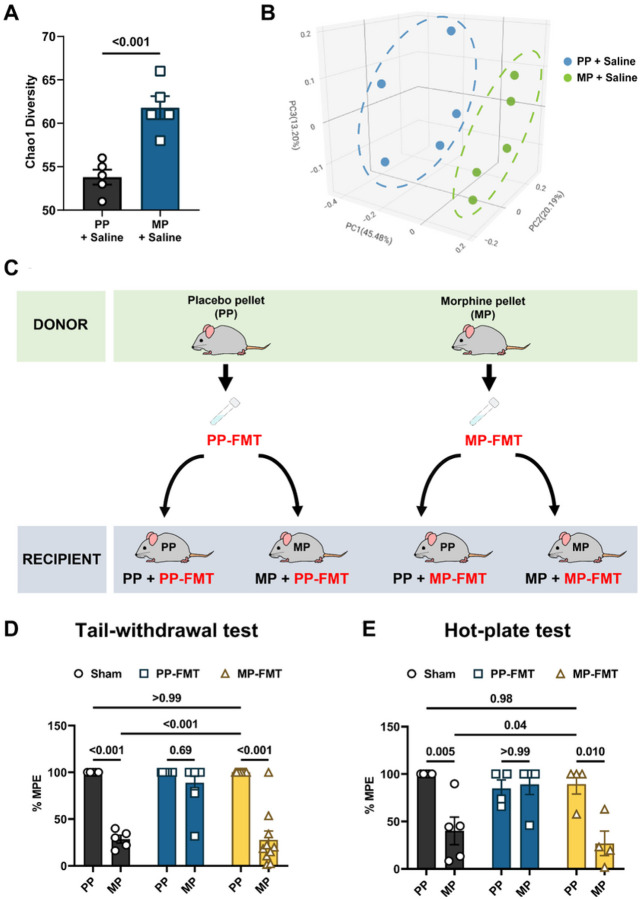
Effect of FMT on the development of tolerance to morphine-induced antinociception. (A) Alpha diversity was assessed in the fecal samples of saline-treated PP and saline-treated MP mice using the Chao1 index. (B) Principal coordinate analysis (PCoA) of the beta diversity of the fecal bacteria in saline-treated PP and saline-treated MP mice was measured with the Bray-Curtis Index. (C) Schematic of FMT administration to PP and MP mice. Tolerance to the 10 mg/kg morphine challenge was assessed in pelleted mice using the (D) warm-water tail-withdrawal and (E) hot-plate tests. Mice were either sham-treated (i.e., no FMT) or administered PP-FMT or MP-FMT. Low %MPE values indicated the development of tolerance. Data in A was analyzed by two-tailed unpaired Student’s t-test. Data in D and E were analyzed by two-way ANOVA, and Tukey’s multiple comparisons test was used for *post hoc* analysis. P values for relevant interactions have been indicated. Data in A, D and E are mean ± SEM, and scatter in A, B, D, and E represents data from individual mice. Sample sizes in A and B are 5 mice per group. Sample sized in D are: 5 (PP-Sham and MP-Sham) and 10 (PP + PP-FMT, MP + PP-FMT, PP + MP-FMT, and MP + MP-FMT) mice. Sample sizes in E are: 5 (PP-Sham and MP-Sham), 4 (PP + PP-FMT), 5 (MP + PP-FMT), and 4 (PP + MP-FMT and MP + MP-FMT) mice.

Evaluation of the alpha diversity (within group diversity) of fecal bacterial communities using the Chao1 Index revealed significantly increased bacterial abundance in the saline-treated morphine-pelleted (MP) animals compared to the saline-treated placebo-pelleted (PP) animals (Fig. 1A). Principal coordinate analysis of the beta diversity (diversity between groups) of the fecal bacteria measured with the Bray-Curtis Index revealed discrete clustering of the saline-treated morphine-pelleted (MP) mice and saline-treated placebo-pelleted (PP) mice (Fig. 1B, Table S1). Further analysis of the bacterial taxa at the phylum level showed contraction of *Firmicutes* and *Actinobacteria*, and expansion of *Bacteroidetes* in saline-treated MP mice (Figure S1). Altogether, the data indicated that chronic morphine treatment altered the composition of the fecal bacteria.

### Fecal microbiota transplants from placebo-pelleted mice inhibited the development of tolerance to morphine-induced antinociception.

Opioid-induced dysbiosis has been implicated in the development of tolerance to opioid-induced antnociception^11^. Since chronic morphine exposure induced dysbiosis, we investigated whether replacing the bacteria of morphine-treated mice with that of control mice using fecal microbiota transplants (FMT) altered the development of tolerance to morphine-induced antinociception (Fig. 1C). A two-way ANOVA analysis of the antinociceptive effect produced by 10 mg/kg morphine in the warm-water tail-withdrawal test revealed a significant FMT x morphine treatment interaction [F (2,44) = 19.36, P< 0.001] (Fig. 1D). Chronic morphine-treated mice responded poorly to the 10 mg/kg morphine challenge compared to PP mice (28.5 ± 4.2 %PE vs. 100 ± 0 %PE in PP mice; Fig. 1D), indicating the development of tolerance. FMT from PP mice (PP-FMT) prevented the development of tolerance to morphine-induced antinociception. 10 mg/kg morphine produced significant antinociception in MP mice treated with PP-FMT (88.9 ± 7.0 %PE; Fig. 1D). Interestingly, FMT from MP mice (MP-FMT) did not produce tolerance to morphine-induced antinociception in PP mice.

A two-way ANOVA analysis of the hot-plate data indicated a significant interaction of FMT x morphine treatment [F (2, 21) = 6.154, P = 0.008] (Fig. 1E). Chronic morphine-treated mice were tolerant to the 10 mg/kg morphine challenge dose in the hot-plate test (40.1 ± 14.6%MPE vs. 100 ± 0%MPE in PP mice; Fig. 1E). Consistent with the warm-water tail-withdrawal test results, PP-FMT prevented the development of tolerance to morphine-induced antinociception in MP mice. MP mice treated with PP-FMT exhibited significant antinociception to 10 mg/kg morphine (89.2 ± 10.8%MPE; Fig. 1E). Thus, replenishing the fecal microbiota of morphine-treated mice prevented the development of tolerance to morphine-induced antinociception in the warm-water tail-withdrawal and hot-plate tests. However, treating PP mice with the microbiota of MP mice did not produce tolerance to morphine-induced antinociception (Figs. 1D and 1E). 10 mg/kg morphine produced significant antinociception in PP mice treated with MP-FMT (Figs. 1D and 1E). These results suggest that dysbiosis is not sufficient for producing tolerance to morphine-induced antinociception and that exposure to chronic morphine is required for the induction of tolerance to morphine-induced antinociception.

### Exposure to chronic morphine reduced endogenous butyrate concentration in the stool.

**Figure 2:**
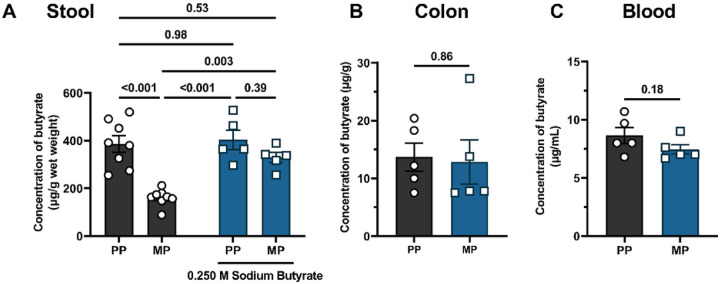
Concentration of endogenous butyrate in stool, colon tissue and blood. The concentration of endogenous butyrate was determined in (A) stool, (B) colon, and (C) blood samples from pelleted mice using GC-MS. Chronic exposure to morphine reduced endogenous butyrate in stool but not in colon or blood. Oral administration of 0.250 M sodium butyrate replenished butyrate levels in the stool samples of morphine-treated mice. Data in A were analyzed using two-way ANOVA with Tukey’s multiple comparisons *post* hoc test. Data in B and C were analyzed using unpaired two-tailed Student’s t-test. P values for comparisons between groups have been indicated. Data are mean ± SEM and scatter represent data from individual mice. Sample sizes for A are: 8 (PP and MP), and 5 (PP + sodium butyrate, and MP + sodium butyrate). Sample sizes for B and C are: 5 (PP and MP).

Butyrate levels are substantially reduced in fecal samples of opioid users ^47^. Furthermore, the phylum *Firmicutes*, which comprises butyrate-producing bacteria^22^, was reduced in chronic morphine-treated mice (Figure S1). Therefore, we analyzed the concentration of endogenous butyrate in fecal samples, colon tissue, and whole blood in chronic morphine-treated mice using GC-MS (Fig. 2). The concentration of butyrate in fecal samples of MP mice was significantly reduced compared to that of PP mice (386.1 ± 34.5 vs. 161.0 ± 12.3, respectively). Daily oral supplementation with sodium butyrate restored butyrate levels in the fecal samples of MP mice (Fig. 2A). Daily butyrate supplementation in placebo mice did not increase butyrate concentrations in the stool, likely due to quorum sensing^23^. We observed no change in the concentration of butyrate in colon tissue [13.7 ±2.5 vs. 12.8 ± 3.8, t (df) = 0.186 (8), P = 0.86] or whole blood [8.6 ± 0.7 vs. 7.5 ± 0.4, t (df) = 1.466 (8), P = 0.18] between PP and MP mice (Figs. 2B and 2C, respectively). These data suggest that in chronic morphine-treated mice, butyrate production is significantly impaired due to altered intestinal microbiota.

### Butyrate administration prevented the development of tolerance to morphine-induced antinociception.

**Figure 3:**
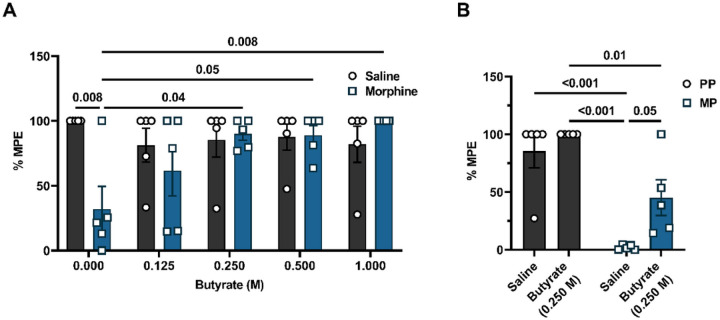
Effect of sodium butyrate on the development of tolerance to morphine-induced antinociception. (A)Mice were treated with increasing doses of sodium butyrate and the dose dependent effect of butyrate on the development of tolerance to morphine-induced antinociception was evaluated in the warm-water tail-withdrawal test. These mice were intermittently treated with escalating saline or escalating doses of morphine saline to induce tolerance to morphine-induced antinociception. (B) The effect of sodium butyrate on the development of tolerance to morphine-induced antinociception was also evaluated in pelleted mice using the warm-water tail-withdrawal test. Data in A and B were analyzed by two-way ANOVA analysis and Tukey’s test was used for comparisons between groups. P values for relevant comparisons have been indicated. Data are expressed as mean ± SEM and scatter represent data from individual mice. Samples sizes in A are 5/dose/group, and sample sizes for B are 5/group.

We next investigated if butyrate administration altered the development of tolerance to morphine-induced antinociception. Morphine was administered intermittently (Fig. 3A) or continuously through subcutaneously implanted pellets (Fig. 3B). Figure 3A shows that chronic morphine-treated mice were tolerant to morphine-induced antinociception when sodium butyrate was not administered. The 10 mg/kg morphine challenge did not produce significant antinociception (32.0 ±17.6% MPE vs. 100.0 ±0.0% MPE in the saline + 0 M butyrate-treated mice). Oral administration of sodium butyrate prevented the development of tolerance to morphine-induced antinociception in a dose-dependent manner. Significant inhibition of tolerance to morphine-induced antinociception was observed starting with the 0.250 M sodium butyrate dose (89.9 ± 5.0% MPE vs. 32.0 ± 17.6% MPE in the morphine+ 0 M butyrate-treated mice; Fig. 3A). A two-way ANOVA analysis comparing % MPE of the 10 mg/kg morphine challenge in saline or chronic morphine-treated animals administered with increasing doses of sodium butyrate revealed a significant butyrate dose x morphine treatment interaction [F (4, 40) = 4.015, P = 0.0008] (Fig. 3A). Consistent with results in Fig. 3A, tolerance to morphine-induced antinociception was observed in MP mice in the warm-water tail-withdrawal assay (2.0 ±0.9% MPE vs. 85.4 ±14.6% MPE in the PP + saline group) and oral administration of 0.250 M sodium butyrate significantly reduced tolerance to morphine-induced antinociception (45.1 ± 15.4% MPE vs. 2.0 ± 0.9% MPE in the MP + saline group, Fig. 3B). A two-way ANOVA analysis of the % MPE of 10 mg/kg morphine in PP or MP mice revealed significant main effects of butyrate treatment [F (1, 16) = 7.373, P = 0.02] and morphine treatment [F (1, 16) = 42.49, P< 0.001] but no interaction effects [F (1, 16) = 1.804, P = 0.20], These data implied that tolerance to morphine-induced antinociception is inhibited by microbial metabolites such as butyrate irrespective of the route of morphine administration or the method of inducing tolerance.

### Butyrate or fecal microbiota transplantation reduced chronic morphine-induced intestinal epithelial barrier disruption.

**Figure 4:**
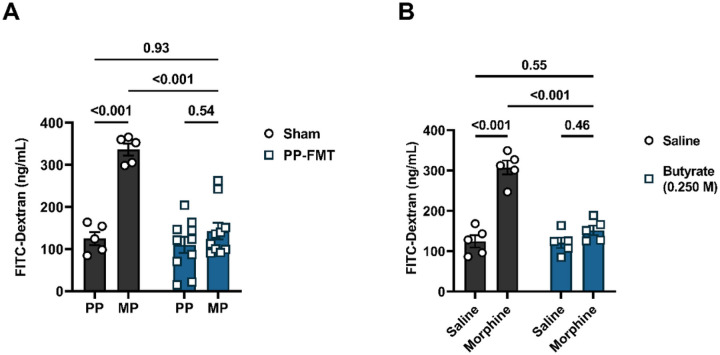
Effect of FMT or butyrate on morphine-induced disruption of epithelial permeability. FITC-dextran was fluorometrically quantified from whole blood samples in morphine-treated mice after (A) FMT or (B) sodium butyrate administration. Data in A and B were analyzed by two-way ANOVA with Tukey’s multiple comparisons test. Relevant P values have been indicated. Data are expressed as mean ± SEM and scatter represent data from individual mice. Sample sizes in A are: 5 (PP-Sham, and MP-Sham) and 10 (PP + PP-FMT, and MP + PP-FMT). Sample sizes in B are 5/group.

Increasing evidence indicates that chronic exposure to morphine results in the disruption of the intestinal epithelium^11,24^. FMT of commensal microbiota and butyrate are known to protect epithelial barrier function through multiple mechanisms, including regulating the permeability of the epithelium^22,25^. Flere, the effect of FMT or sodium butyrate on the transepithelial permeability of chronic morphine-treated mice was assessed using FITC-dextran (Fig. 4). We detected significantly higher serum levels of FITC-dextran in chronic morphine-treated mice compared to placebo (Fig. 4A) or saline (Fig. 4B) controls. Since the presence of FITC-dextran in serum indicates enhanced intestinal permeability, these results implicate that chronic morphine exposure caused an increase in passive diffusion by disrupting the intestinal epithelium. PP-FMT (Fig. 4A) or sodium butyrate (Fig. 4B) significantly reduced serum levels of FITC-dextran in chronic morphine-treated mice (Fig. 4). No significant difference in serum levels of FITC-dextran was observed between PP mice and FMT-treated MP mice (Fig. 4A) or between saline and chronic morphine + sodium butyrate-treated mice (Fig. 4B). These data demonstrate that the intestinal epithelium remained intact despite chronic morphine treatment.

### Butyrate or FMT prevented morphine-induced disruption of the antimicrobial activity of the small intestinal epithelium.

**Figure 5:**
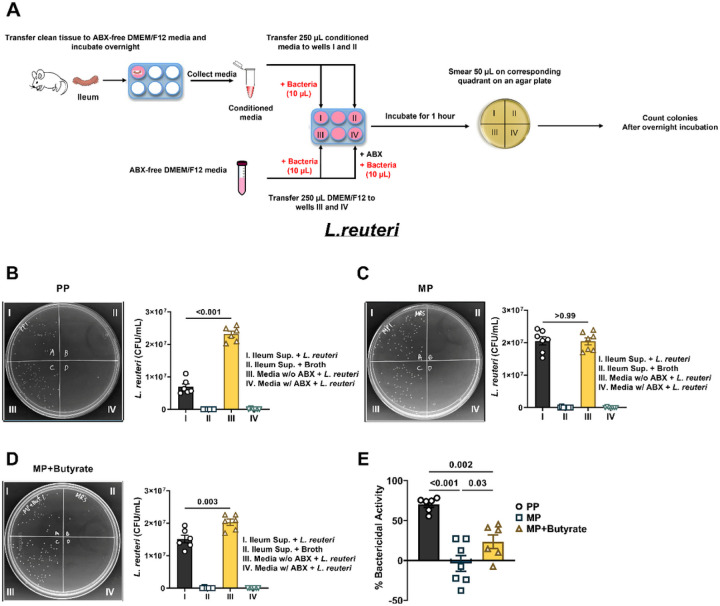
Effect of morphine and sodium butyrate on the activity of the ileum against *L.reuteri*. (A) Schematic of the antibacterial activity assay protocol. Each agar plate is divided into quadrants and in quadrant I ileum-derived conditioned media incubated with bacteria was smeared. In quadrant II ileum conditioned media containing only nutrient broth and no bacteria was applied; in quadrant III antibiotic-free DMEM/F12 media incubated with bacteria was smeared; and in quadrant IV antibiotic-containing DMEM/F12 media incubated with bacteria was applied. Conditioned media obtained from the ileum of (B) PP mice, (C) MP mice or (D) MP mice treated with 0.250 M sodium butyrate (MP + Butyrate) was used to evaluate the antibacterial activity of the ileum against the Gram-positive bacteria, *L.reuteri*. Representative images of *L.reuteri* growing on agar plates have been shown in B-D. Figure E is the normalized data of the %bactericidal activity of the ileum supernatants from PP MP and MP + butyratemice. Data in B-D were analyzed by repeated-measures one-way ANOVA and Tukey’s multiple comparisons test was used for *post hoc* analysis. Data in E was analyzed by one-way ANOVA and Holm-Sidak’s multiple comparisons test was used for *post hoc* analysis. P values for relevant comparisons have been indicated. All data are mean ± SEM and scatter represent data from samples from individual mice. Sample sizes are 6, 7, and 6 per group for B, C, and D, respectively. Sample sizes for E are: 6 (PP), 7 (MP) and 6 (MP + butyrate).

AMPs contribute to the innate immune response of the intestinal epithelium by maintaining the intestinal microbiota and preventing colonization of the epithelium by pathogenic strains^21^. It is unclear if the altered antimicrobial activity of the intestinal epithelium contributes to opioid-induced dysbiosis. Therefore, we investigated whether chronic morphine treatment perturbed the antibacterial activity of the ileum and if sodium butyrate or FMT inhibited morphine’s effects (Fig. 5A). We first examined antimicrobial activity against the prototypical Gram-positive bacteria, *L.reuteri (*Fig. 5). Incubation of *L.reuteri* with conditioned media from the ileum of PP mice resulted in significantly reduced colonies on the agar plate compared to when the bacteria were incubated with antibiotic-free nutrient media (6.9 × 10^6^ ± 9.4 × 10^5^ CFU/mL vs. 2.3 × 10^7^ ± 9.2 × 10^5^ CFU/mL, respectively; Fig. 5B), indicating that the conditioned media exhibited activity against Gram-positive bacteria. In comparison, conditioned media prepared from the ileum of MP mice yielded similar colony numbers to when the bacteria were incubated in antibiotic-free nutrient media (2.1 × 10^7^ ± 1.3 × 10^6^ CFU/mL vs. 2.0 × 10^7^ ± 1.1 × 10^6^ CFU/mL, respectively; Fig. 5C), suggesting that the antibacterial activity of the conditioned media decreased after chronic morphine treatment. Interestingly, the antibacterial activity of the ileum-conditioned media was somewhat restored upon treatment with sodium butyrate (Fig. 5C). The conditioned media from the ileum of mice treated with morphine and sodium butyrate yielded fewer colonies compared to when the bacteria were incubated with antibiotic-free nutrient media (1.5 × 10^7^ ± 1.2 × 10^6^ CFU/mL vs. 2.0 × 10^7^ ± 9.6 × 10^5^ CFU/mL, respectively; Fig. 5D). A comparison of the normalized data in Fig. 5E revealed that chronic morphine treatment significantly reduced bactericidal activity of the ileum against Gram-positive bacteria, which was partially restored with oral butyrate treatment (70.1 ± 3.6% in PP mice vs. −3.6 ± 9.9% in MP mice vs. 23.6 ± 8.6% in MP + butyrate-treated mice).

**Figure 6:**
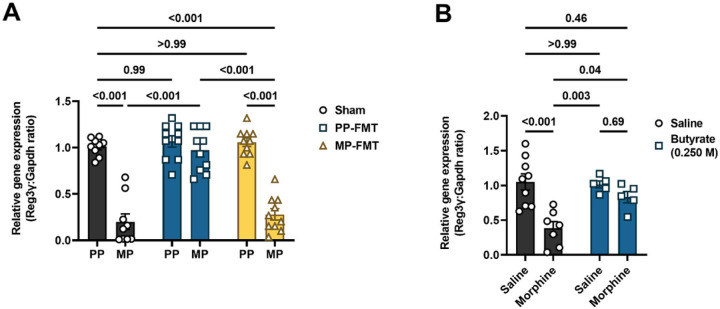
Effect of morphine, FMT and sodium butyrate on the expression of Reg3γ in the ileum. The expression of the AMP Reg3γ, which is active against Gram-positive bacteria, was measured in the ileum in chronic morphine-treated mice administered (A) FMT or (B) 0.250 M sodium butyrate. Data in A and B were analyzed by two-way ANOVA analysis and Tukey’s *post hoc* test was used for multiple comparisons between groups. Relevant P values are indicated. Data are mean ± SEM and scatter represent data from ileum tissues of individual mice. Sample sizes for A are 9 (sham groups), 10 (PP-FMT groups), and 10 (MP-FMT groups). Sample sizes for B are 9 (Saline + Saline), 7 (Saline + Morphine), 5 (Butyrate + Saline), and 5 (Butyrate + Morphine).

The REG3 family of antimicrobial peptides is highly expressed in the small intestine. Murine REG3γ, and its human analog, REG3α, have been reported to exhibit activity against Gram-positive bacteria^26,27^. Since REG3γ is transcriptionally regulated, we investigated whether morphine altered the expression of the Reg3γ gene in the ileum and if FMT of commensal microbiota comprising butyrate-producing bacteria (Fig. 6A) or direct administration of exogenous butyrate (Fig. 6B) inhibited morphine effects. Reg3γ expression was significantly reduced after chronic morphine treatment, i.e., in MP mice compared to PP controls (Fig. 6A) and morphine-injected mice compared to saline controls (Fig. 6B). Interestingly, FMT of from placebo-pelleted mice (PP-FMT) prevented the downregulation of Reg3γ in morphine-pelleted mice. However, FMT from morphine-pelleted mice (MP-FMT) produced no effect on Reg3γ expression in placebo-pelleted mice, and Reg3γ expression in the Ileum of these mice remained significantly upregulated (Fig. 6A). The absence of the effect of MP-FMT on Reg3γ expression indicated that the antimicrobial response of the epithelial barrier remained intact and that chronic treatment with morphine was necessary for inducing barrier dysfunction. Consistent with the results of PP-FMT treatment on Reg3γ expression in morphine-treated mice, oral administration of sodium butyrate also prevented morphine-induced downregulation of Reg3γ in the ileum (Fig. 6B). Sodium butyrate did not alter the expression of Reg3γ in saline-treated control mice (Fig. 6B). The absence of an effect could be due to quorum sensing by the autochthonous microbiota^23^. The data indicated that the commensal microbiota and/or butyrate regulate Reg3γ transcription to reverse chronic morphine-induced effects.

**Figure 7:**
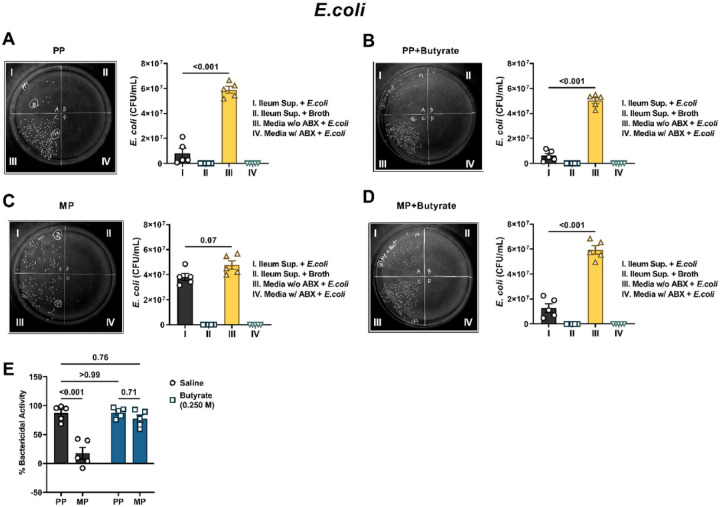
Effect of morphine and sodium butyrate on the activity of the ileum against *E.coli*. Conditioned media obtained from the ileum of (A) PP mice, (B) PP mice treated with 0.250 M sodium butyrate (PP + butyrate), (C) MP mice or (D) MP mice treated with 0.250 M sodium butyrate (MP + Butyrate) was used to evaluate the antibacterial activity of the ileum against the Gram-negative bacteria, *E.coli*. Representative images of *E.coli* growing on agar plates have been shown in A-D. Each agar plate is divided into quadrants and in quadrant I ileum-derived conditioned media incubated with bacteria was smeared. In quadrant II ileum conditioned media containing only nutrient broth and no bacteria was applied; in quadrant III antibiotic-free DMEM/F12 media incubated with bacteria was smeared; and in quadrant IV antibiotic-containing DMEM/F12 media incubated with bacteria was applied. Figure E is the normalized data of the %bactericidal activity of the ileum supernatants from PP PP + butyrate, MP and MP + butyrate mice. Data in A-D were analyzed by repeated-measures one-way ANOVA and Tukey’s multiple comparisons test was used for *post hoc* analysis. Data in E was analyzed by two-way ANOVA and Tukey’s multiple comparisons test was used for *post hoc* analysis. P values for relevant comparisons have been indicated. All data are mean ± SEM and scatter represents data from samples from individual mice. Sample sizes are 5 per group for A-D. Sample sizes for E are: 5/group.

Next, we tested the activity of the ileum supernatants against the prototypical Gram-negative bacteria, *E.coli* (Fig. 7). Bacterial colony counts were significantly diminished when *E.coli* were incubated with conditioned media from the ileum of PP mice instead of antibiotic-free nutrient media (5.9 × 10^7^ ± 2.7 × 10^6^ CFU/mL vs. 8.1 × 10^6^ ± 4.0 × 10^6^ CFU/mL, respectively; Fig. 7A), indicating that the ileum-derived conditioned media exerted substantial antibacterial activity against Gram-negative bacteria. Sodium butyrate did not affect the antibacterial activity of the ileum-derived conditioned media. *E.coli* colony counts were substantially reduced when incubated with the conditioned media from the ileum of PP mice treated with sodium butyrate instead of antibiotic-free DMEM/F12 media (5.0 × 10^7^ ± 2.3 × 10^6^ CFU/mL vs. 6.2 × 10^6^ ± 1.9 × 10^6^ CFU/mL, respectively Fig. 7B). Figure 7C shows the reduced antibacterial activity of ileum supernatants obtained from chronic morphine-treated mice. Incubation of *E.coli* with conditioned media from the ileum of MP mice resulted in a non-significant reduction in colony numbers compared to when the bacteria were incubated with antibiotic-free nutrient media (3.8 × 10^7^ ± 2.9 × 10^6^ CFU/mL vs. 4.8 × 10^7^ ± 3.4 × 10^6^ CFU/mL, respectively). Conditioned media derived from the ileum of chronic morphine-pelleted mice treated with sodium butyrate exerted significant antibacterial activity (Fig. 7D). *E.*coli colony counts were substantially larger when antibiotic-free nutrient media was used instead of conditioned media from the ileum of sodium butyrate-treated chronic morphine-pelleted mice (5.9 × 10^7^ ± 3.4 × 10^6^ CFU/mL vs. 1.3 × 10^7^ ± 3.4 × 10^6^ CFU/mL, respectively; Fig. 7D). A comparison of the normalized data in Fig. 7E revealed that the % Bactericidal activity of morphine-treated ileum media was significantly reduced compared to that from PP mice (17.5± 10.0% vs. 87.1 ± 6.0%, respectively). While oral administration of sodium butyrate did not alter the antibacterial activity of the ileum from PP mice, it significantly increased the bactericidal activity of the ileum of MP mice (87.9 ± 3.9% and 77.3 ± 6.4%, respectively; Fig. 7E). These data indicated that chronic treatment with morphine inhibited the homeostatic activity of the ileum from fighting against Gram-positive and Gram-negative bacteria and that oral administration of sodium butyrate can prevent these effects.

### Oral butyrate administration altered the fecal bacterial composition in morphine-treated mice.

Analysis of the Chao1 Index of alpha diversity revealed that the bacterial abundance in butyrate-treated MP mice was diminished compared to saline-treated MP mice and was comparable to saline-treated PP animals. Oral butyrate administration did not alter the Chad diversity of the fecal bacteria of PP mice (Figure S2A). Evaluation of the beta diversity using the Bray-Curtis Index revealed distinct clustering of butyrate-treated MP mice and saline-treated MP mice (Table S1; Figure S2B). PERMANOVA analysis indicated a significant difference in the beta diversity of butyrate-treated MP mice and saline-treated PP mice (Table S1; Figure S2C). PERMANOVA analysis did not reveal differences in the beta diversity of saline-treated PP mice and butyrate-treated PP mice (Table S1; Figure S2D). Altogether, these data indicated that butyrate altered the fecal microbial composition of chronic morphine treated mice.

### Naloxone antagonized the morphine-induced decrease in the antimicrobial activity of the ileum.

**Figure 8:**
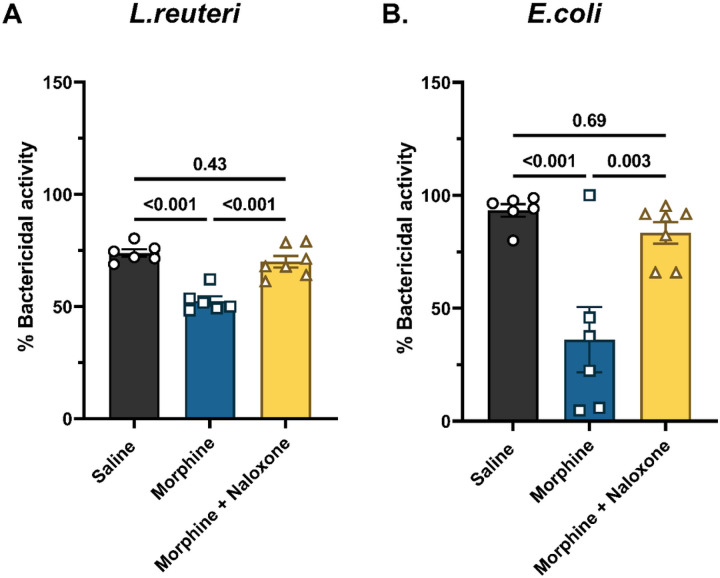
Effect of naloxone on morphine-induced decrease in antibacterial activity of the ileum. The effect of the non-selective opioid receptor antagonist, naloxone, was evaluated on the inhibitory effect of morphine on the antibacterial activity of the ileum against (A) *L.reuteri*, and (B) *E.coli*. Data are normalized %bactericidal activity and presented as mean ± SEM. Scatter represents data from ileum-derived conditioned media from individual mice. Data were analyzed by one-way ANOVA and Tukey’s multiple comparisons test was used for *post hoc* analysis. P values for relevant comparisons have been indicated. Sample sizes in A and B are 6, 6, and 7 for saline, morphine and morphine + naloxone groups, respectively.

We next tested if the inhibitory effect of morphine on the antibacterial activity of the ileum was opioid receptor-mediated. Morphine treatment reduced the activity of the ileum supernatants against *L*.reuteri (52.5 ± 2.1% in morphine-treated vs. 73.8 ± 1.7% in saline-treated; Fig. 8A) and against *E*.coli (36.1 ± 14.5% in morphine-treated vs. 93.3 ± 2.8% in saline-treated) (Fig. 8B). Concomitant treatment with naloxone, a non-selective opioid receptor antagonist, prevented morphine effects (*L.reuteri*: 69.9 ± 2.6%; *E.coli*: 83.4 ± 4.8%; Fig. 8), thus indicating that the effect of chronic morphine exposure on the antibacterial activity of the ileum is opioid receptor-mediated.

## DISCUSSION

In this paper we show that the antimicrobial activity of the gastrointestinal tract is markedly reduced in a morphine-dependent mouse model. Chronic morphine inhibits the expression of the antimicrobial peptide, Reg3γ, that is prevented by fecal transplant from morphine-naïve mice or by the short chain fatty acid, butyrate. Supplementing the enteric microbiome of morphine-treated mice with butyrate or the fecal microbiota of morphine-naive mice attenuated tolerance to morphine-induced antinociception. Altogether, the results indicate that morphine-induced disruption of the antimicrobial activity of the intestinal epithelium contributes to enteric dysbiosis and impacts the development of antinociceptive tolerance.

The intestinal epithelium restricts pathogenic strains from colonizing intestinal tissue and entering systemic circulation through multiple contingencies that maintain spatial segregation between the intestinal epithelium and the gut bacteria. For example, tight junction proteins between adjacent intestinal epithelial cells limit paracellular transport between the apical and basolateral membranes^28^. Intestinal epithelial cells facilitate the secretion of IgA, produced by immune cells in the underlying lamina propria, into the lumen^19^. Goblet cells produce mucus which entraps bacteria and keeps them at bay. Enterocytes and Paneth Cells within intestinal crypts secrete AMPs into the lumen ^21^. The antimicrobial activity of the intestinal epithelium is reduced, and systemic translocation of luminal bacteria is observed if Paneth cells are damaged, or their activity is disrupted ^20^. Similar effects were observed in the present study in mice exposed to chronic morphine. Here, we observed that repeated exposure to morphine, whether through intermittent injections or subcutaneously implanted continuous-release pellets, reduced the antimicrobial activity of the intestinal epithelium. Activity against both Gram-positive and Gram-negative bacteria was reduced. Specifically, the antimicrobial c-type lectin, Reg3γ, was downregulated in chronic morphine-treated mice. Reg3γ promotes host-bacteria segregation, and consequently, Reg3γ^−/−^ mice exhibit altered mucus distribution, increased colonization of the intestinal epithelium by mucosa-associated bacteria, bacterial translocation, and elevated inflammatory responses in the intestine^29,30^. Naloxone, a nonselective opioid receptor antagonist, prevented morphine effects on the antimicrobial activity of the ileum against Gram-positive and Gram-negative bacteria, indicating that this effect was opioid receptor-mediated. In addition to the perturbed antimicrobial activity of the intestinal epithelium, chronic morphine exposure altered the composition of the gut bacteria. Thus, these data suggest that impaired ability to neutralize bacteria contributes to opioid-induced intestinal dysbiosis and predisposes to systemic translocation of luminal bacteria via disruption of the epithelial barrier.

μ-opioid receptors are not detected in intestinal epithelial cells; they are primarily expressed in neurons^1,31^. Thus, altered activity of neurons might form the basis for opioid-induced intestinal barrier dysfunction and the resultant dysbiosis. There is increasing evidence that enteric neurons can modulate the response of the intestinal epithelial barrier to gut microbiota. Chemogenetic activation of cholinergic enteric neurons altered the intestinal transcriptome, including the expression of genes responsible for mucosal immunity and antimicrobial responses, and produced effects on the gut microbiome and metabolome^32^. Approximately half of the μ-opioid receptor-expressing neurons in the enteric nervous system are cholinergic^31,33^. A subset of μ-opioid receptor-containing neurons also expresses the inhibitory neurotransmitter, vasoactive intestinal peptide (VIP)^31,33^. VIPergic neurons have been shown to regulate the activity of intestinal epithelial cells via crosstalk with innate lymphoid cells, ILC2, and ILC3^34–36^. Morphine is known to modulate the excitability of enteric neurons by altering the activity of voltage-gated ion channels^37–40^. Morphine can also alter the expression of IL-18 in enteric neurons via opioid receptors^33^. IL-18 from enteric neurons, but not immune or epithelial cells, is necessary to produce AMPs, which protect against bacterial infiltration of the intestinal epithelium^41^. Thus, disrupting the homeostatic AMP production through effects on enteric neurons might be a potential mechanism by which morphine inhibits the intestinal barrier function. The intestinal mucosa also receives innervation from extrinsic nerve fibers of the vagus, spinal afferents, and sympathetic neurons ^42^; these neurons also play an essential role in maintaining the intestinal epithelial barrier. Gut-innervating nociceptive dorsal root ganglia neurons can direct goblet cells to produce mucus and maintain the epithelial barrier^43^. These neurons can also regulate the density of specialized antigen-sampling microfold cells in the gut-associated lymphoid tissue of the Peyer’s patches. Breakdown of this crosstalk predisposes the intestinal mucosa to invasion by pathogenic microbes^44^. Like with enteric neurons, chronic exposure to morphine significantly alters the excitability of nociceptive dorsal root ganglia neurons^11,12^. Studies are required to investigate the contribution of gut-innervating extrinsic neurons versus enteric neurons in the mechanism of morphine-induced disruption of the intestinal barrier.

Replacing the dysbiotic gut flora with “normal” flora by FMT is clinically utilized for treating several intestinal pathologies^45–48^. Banerjee et al.^14^ reported that FMT from control mice prevented morphine-induced dysbiosis and disruption of the intestinal epithelial barrier. Consistent with these findings, in the present study, PP-FMT prevented morphine-induced epithelial permeability and downregulation of Reg3γ, indicating improved epithelial barrier function. Metabolomic analysis of the fecal samples of PP and MP mice revealed reduced butyrate levels in chronic morphine-treated mice. Similar results have been noted in the stool samples of human patients exposed to opioids^7^. Consistent with prior findings^11^, the abundance of the phylum *Firmicutes*, which comprises butyrate-producing bacteria^22^, was diminished in chronic morphine-treated mice. The depletion of *Firmicutes* might explain low fecal butyrate levels in chronic morphine-treated mice.

Butyrate is well-known for its positive effects on host-gut microbiota interactions as it engages pathways that regulate epithelial permeability, inflammation, and immune function^49^. Butyrate administration protected against the morphine-induced increase in epithelial permeability. This effect of butyrate could be due to its actions on epithelial tight junction proteins, such as occludin and zonula occluden protein-1^49^, which are downregulated in the ileum of chronic morphine-treated mice^24^. Additionally, butyrate conserved the antimicrobial activity of the intestinal epithelium and inhibited morphine-induced downregulation of Reg3γ. SCFAs, including butyrate, have been reported to induce the expression of several antimicrobial peptides, including Reg3γ^50–56^. The improved epithelial barrier function of the intestinal epithelium in chronic morphine-treated mice supplemented with butyrate might explain the shifts in bacterial community. Several mechanisms underlying the mitigating effects of butyrate on the epithelial barrier have been proposed, such as the activation of G-protein-coupled receptors (GPR41, GPR43, and GPR109A) and transcriptional regulation of genes by histone deacetylase inhibition and histone acetyltransferase activation^57–60^. It is important to note that G-protein-coupled receptors for SCFAs are found not only on epithelial cells but also expressed by enteric neurons, and autonomic and somatosensory ganglia^61–63^. Hence, the effects of butyrate on the epithelial barrier could also be modulated, in part, by direct actions on neurons.

Oral butyrate administration reduced the alpha diversity of the fecal microbiota to placebo levels. However, the beta diversity remained significantly different. The intestinal epithelium maintains the composition of the enteric bacteria through multiple mechanisms, including secretion of AMPs by Paneth cells and IgA by lamina propria immune cells ^19^. Morphine exposure has been previously reported to reduce IgA production by the gut-associated lymphoid tissue through the downregulation of TGF-β^64^. Further studies are required to investigate the effect of butyrate on IgA production in morphine-treated mice.

Several studies have reported CNS effects of microbial-derived butyrate or orally administered butyrate, including in substance use disorders^65,66^. In the present study, oral administration of PP-FMT or sodium butyrate to chronic morphine-treated mice attenuated the development of tolerance to morphine-induced antinociception. The inhibition of tolerance was dependent on the dose of sodium butyrate. Although SCFAs can cross the blood-brain barrier^76–79^, approximately 95% of orally administered butyrate is absorbed locally in the intestine and extremely low concentrations of oral butyrate are detected in systemic circulation or brain^67–69^. Thus, the effect of orally administered sodium butyrate on the development of tolerance to morphine-induced antinociception is most likely through peripheral processes mediating the crosstalk between the gut and the brain. Accumulating evidence indicates that gut microbes, through their products, such as SCFAs, can modulate neural circuits that relay information between the PNS and the CNS^70–72^. A recent study showed that eliminating gut bacteria increased the expression of c-Fos, a marker of neuronal activity, in gut-innervating neurons that connect the brainstem sensory nuclei to the sympathetic ganglia, but colonization of mice with butyrate-producing bacteria or feeding butyrate reduced c-Fos expression ^70^. Furthermore, eliminating enteric bacteria with antibiotics prevented the development of morphine tolerance in nociceptive dorsal root ganglia neurons^11,12^. Nociceptive dorsal root ganglia neurons transduce noxious stimuli from the periphery to the CNS and have been implicated in the induction of tolerance to morphine-induced antinociception^73,74^. Treating naive dorsal root ganglia neurons with gut-derived mediators from chronic morphine-treated mice induced morphine tolerance^12^, which was attenuated when cells were incubated with gut-derived mediators from chronic morphine-treated mice exposed to antibiotics^12^.

In the present study, FMT from placebo-pelleted mice attenuated tolerance in chronic morphine-treated mice. The reverse was not observed when placebo-pelleted mice received FMT from morphine-pelleted mice. Placebo-pelleted mice treated with FMT from morphine-pelleted mice did not develop tolerance to morphine. These results were inconsistent with a previous report from Zhang et al.^16^ that noted the induction of tolerance to morphine-induced antinociception in germ-free mice treated with FMT from chronic morphine-treated animals^16^. Germ-free mice exhibit significant developmental deficits, especially in the gut-associated immune system^75^; consequently, the microbiota could disrupt the epithelial barrier without resistance and activate downstream events that result in the development of tolerance. Specific pathogen-free mice have an intact barrier function that maintains spatial segregation between the intestinal epithelium and the bacteria contained within the FMT of morphine-pelleted mice. In the present study, placebo-pelleted mice treated with FMT from morphine-pelleted mice exhibited reduced epithelial permeability and intact Reg3γ expression comparable to that in sham-treated placebo mice. Placebo-pelleted mice treated with FMT from morphine-treated animals also exhibited reduced IL-17 levels compared to morphine-pelleted mice treated with FMT from morphine-treated animals^14^. Elevated IL-17 levels have been previously reported in chronic morphine-treated mice, and neutralization of IL-17 protected epithelial barrier function and prevented opioid-induced dysbiosis^15^. Therefore, our results that MP-FMT does not disrupt epithelial barrier function and induce tolerance to morphine-induced antinociception in placebo-pelleted mice indicate that dysbiosis alone is insufficient for downstream events and that morphine-induced disruption of the homeostatic functions of the intestinal epithelium is essential for the development of tolerance.

## LIMITATIONS OF THE PRESENT STUDY

In the present study, there was a significant reduction in the antimicrobial activity of the intestinal epithelium of chronic morphine-treated mice against Gram-negative bacteria. However, the identity of specific AMPs active against Gram-negative bacteria was not determined. One possible candidate could be Reg3β, an isoform of Reg3γ, which is inducible and exhibits activity against Gram-negative bacteria^76^. Like Reg3γ, Reg3β limits mucosa-associated bacteria, and ablation of Reg3β results in dysbiosis, bacterial colonization of Peyer’s patches, systemic translocation of gut bacteria, and reduced survival of mice infected with pathogenic bacteria^77–79^.

A second limitation of the current study is that only male mice were investigated. Studies in humans and rodents have reported sex as a biological variable influencing opioid-induced antinociception^80^. Additionally, sexual dimorphism in the composition of gut bacteria has been noted in laboratory animals and humans, and studies have shown that sex hormones actively influence the gut microbiome^81^.

## CONCLUSION

The intestinal epithelium plays a critical role in controlling the composition of the intestinal microbiota and helps maintain a symbiotic relationship with commensal bacteria. Here, we report that chronic treatment with morphine reduced the antimicrobial activity of the intestinal epithelium via opioid receptors, implicating a potential mechanism underlying opioid-induced dysbiosis. Preventing morphine-induced disruption of the epithelial barrier function with FMT or sodium butyrate inhibited the development of tolerance to antinociception. Finally, dysbiosis alone is insufficient for inducing tolerance to morphine-induced antinociception and disruption of the intestinal epithelium is required for the development of tolerance. In conclusion, our results implicate a mechanism by which morphine disrupts homeostasis in the microbiota-gut-brain axis.

## MATERIALS AND METHODS

### Animals

Male Swiss Webster mice (Harlan Sprague Dawley, Inc. Fredrick, MD, USA) six-eight weeks old, weighing 25–30 g, were housed five to a cage with *ad libitum* access to food and water in animal care quarters maintained under a 12-hour light/dark cycle (lights on from 7 am to 7 pm). Animals were randomly assigned to control and treatment groups. All animal procedures were in accordance with the protocols reviewed and approved by the Institutional Animal Care and Use Committee at Virginia Commonwealth University (VCU IACUC). Results of the animal experiments were reported in accordance with the recommendations of the ARRIVE 2.0 guidelines.

#### Test Drugs:

##### Morphine:

1. 75-mg morphine or placebo pellets, obtained from the National Institute on Drug Abuse (NIDA, Bethesda MD), were aseptically implanted in the subcutaneous cavity on the dorsum under isoflurane (2.5%) anesthesia as described previously^11^. Mice were allowed to recover in their home cages. On test day 7, the mice were subjected to antinociceptive response experiments (Figs. 1 and 3B); used for fecal microbiome analysis (Figs. 1, S1, and S2); for harvesting blood, stool, and colon tissue for evaluating butyrate concentration (Fig. 2); for measuring intestinal epithelial permeability (Fig. 4A); in antibacterial activity experiments (Figs. 5 and 7); or for evaluating Regenerating islet-derived 3 gamma (Reg3γ) gene expression in the ileum (Fig. 6A) as described in the subsequent methods sections. Each animal was used only once.
2. Morphine sulfate (National Institute on Drug Abuse Drug Supply Program, Bethesda, MD) was diluted in saline to 1, 2, 4, and 8 mg/mL. Mice were injected intraperitoneally twice daily with saline or increasing doses of morphine as follows: Day 1–20 mg/kg morphine, Day 2–40 mg/kg morphine, Day 3–40 mg/kg morphine, and Day 4–80 mg/kg morphine. Mice were used on test day 5 in the warm-water tail-withdrawal experiment in Fig. 3A, for evaluating intestinal epithelial permeability in Fig. 4B, for measuring the expression of Reg3γ in Fig. 6B, and in antibacterial activity experiments in Fig. 8 as described in the following methods. Each animal was used only once.

Sodium butyrate: Sodium butyrate (ThermoFisher Scientific, Waltham, MA) was prepared in saline at concentrations of 0.125, 0.250, 0.500, and 1.000 M and administered twice daily by oral gavage. For the dose-response experiment in Fig. 3A, mice injected with ramping doses of morphine (as described above) were orally administered saline or different concentrations of sodium butyrate (0.125, 0.250, 0.500, or 1.000 M) for four days. Antinociception was measured on day 5 using the warm-water tail-withdrawal test described below. In all subsequent experiments, 0.250 M sodium butyrate or its vehicle, saline, was administered twice daily through oral gavage. The number of treatments with sodium butyrate was contingent on the duration of exposure to morphine, such that mice subcutaneously implanted with pellets were administered 0.250 M sodium butyrate for six days (Figs. 3B, 5, 6B, 7, and S2), and mice injected with ramping doses of morphine received 0.250 M sodium butyrate for four days (Figs. 3A, 4B, and 6B).

Naloxone FHCI: Naloxone FHCI (Sigma-Aldrich, St. Louis, MO) was prepared in saline at concentrations of 0.2, 0.4, and 0.8 mg/mL and injected intraperitoneally twice daily at escalating doses 10 minutes before the administration of morphine sulfate in the following manner: Day 1–2 mg/kg naloxone, Day 2–4 mg/kg naloxone, Day 3–4 mg/kg naloxone, and Day 4–8 mg/kg naloxone. The doses of naloxone were 1/10th of the doses of morphine sulfate. Ileum tissue was collected on day 5 for use in the antibacterial activity assay in Fig. 8.

###### Fecal Microbiota Transplant (FMT):

Fresh fecal pellets (100 mg) from placebo or morphine-pelleted mice were collected on day 7 and suspended in 1.2 mL of cold (4°C) phosphate-buffered saline (PBS) containing 10% glycerol. The suspension was homogenized and then centrifuged at 800xg for three minutes. The supernatant was transferred to a separate tube and stored at −80°C. The concentration of total bacteria was determined by measuring optical density (OD), such that OD = 0.5 represented 1×10^8^ cells. 100 μL of the fecal supernatant (1 × 10^9^ cells/dose) was then administered twice daily for six days to recipient mice groups via oral gavage according to the following scheme: 1. Placebo-pelleted mice that did not receive fecal microbiota transplants (PP-Sham), 2. Morphine-pelleted mice that did not receive fecal microbiota transplants (MP-Sham), 3. Placebo-pelleted mice that received fecal microbiota from placebo-pelleted donor mice (PP + PP-FMT), 4. Morphine-pelleted mice that received fecal microbiota from placebo-pelleted donor mice (MP + PP-FMT), 5. Placebo-pelleted mice that received fecal microbiota transplants from morphine-pelleted donor mice (PP+ MP-FMT), and 6. Morphine-pelleted mice that received fecal microbiota transplants from morphine-pelleted donor mice (MP + MP-FMT) (Fig. 1C).

###### Antinociceptive response tests:

The warm-water tail-withdrawal and hot-plate assays were used in the present study. In the warm-water tail-withdrawal test, the distal 1/3 tail was immersed in a water bath at 56°C. The latency to withdraw the tail from the warm water was recorded. A maximum cutoff of 10 seconds was set to prevent tissue damage. On test day 7 in pelleted mice (Figs. 1D and 3B) and test day 5 in injected mice (Fig. 3A), baseline responses were recorded, following which the mice received acute morphine (10 mg/kg s.c.). 25 minutes later, tail-flick latencies were recorded to test for the development of tolerance to the 10 mg/kg morphine challenge. Antinociception induced by 10 mg/kg morphine was quantified as the percentage of maximum possible effect (%MPE), such that: %MPE = [(challenge latency – baseline latency) / (Maximum cutoff – baseline latency)] x 100.

In the hot-plate assay, individual mice were placed on a Syscom Model 35D hot-plate set at 56°C, and the latency to lick their hind paw or jump was recorded. A maximum cutoff of 30 seconds was set to prevent tissue damage. Baseline responses were recorded on test day 7 in pelleted mice (Fig. 1E), following which the mice were injected with acute morphine (10 mg/kg s.c.). 25 minutes later, hot-plate responses were measured again to test for the development of tolerance to the acute morphine challenge.

Antinociception induced by 10 mg/kg morphine was quantified as %MPE as described above.

###### Detection of butyrate in blood, colon, and stool:

Blood, colon tissue samples, and fecal material were collected from morphine or placebo-pelleted mice on day 7. Samples were immediately homogenized 1:4 with deionized water and stored at −30°C. Seven-point calibration curves of 10–1000 μg/g butyrate (Sigma-Aldrich, St. Louis, MO), a butyrate-free control, and a negative control free of butyrate and the internal standard (ISTD) were prepared. Butyrate was extracted and analyzed using a modified previously published method^82^. In brief, 100 μL of methanol containing 20 μg butyrate-1,2-^13^C_2_ (Sigma-Aldrich, St. Louis, MO), the ISTDs, was added to 0.20 g aliquots of each calibrator, control, or specimen except the negative control. Samples were mixed for five minutes, centrifuged for 30 minutes, and then left for 30 minutes at 4°C. 100 μL of the clear supernatant was transferred into a new tube and washed with 100 μL propyl formate. Samples were mixed for five minutes and centrifuged for 30 minutes before transferring 50 μL of the organic layer to GC vials for analysis. Gas chromatography-mass spectrometer (GC-MS) analysis was performed on a Shimadzu GC/MS-QP2020 NX Single Quadrupole GC-MS (Shimazu, Kyoto, Japan) controlled by GCMS solution software (Shimadzu, Kyoto, Japan). Chromatographic separation was performed using a ZB-FFAP column, 30 m × 32mm, 0.25 pm (Phenomenex, Torrance, CA). A sample volume of 2 μL was injected in splitless mode with an injector temperature of 200°C. The carrier gas was Helium with a 2 mL/minute flow rate. The initial oven temperature of 55°C was held for four minutes, then ramped to 130°C at 50°C/minute and held for 3.7 minutes. Finally, the temperature was raised to 250°C at 30°C/minute and held for two minutes. Linear regression of the peak area of ratios of the quantification ion for butyrate (72 *m/z*) and the ISTD quantification ion (75 *m/z)* was used to construct the calibration curves. For each analytical run, the coefficient of determination (r^2^) was higher than 0.996. The concentrations of each calibrator were determined to be within ± 20% of their expected concentration.

### Intestinal permeability assay

On the test day, i.e., day 7 in pelleted mice and day 5 in intermittently injected mice, animals were orally gavaged with FITC-conjugated dextran (100 mg/ml in PBS, Sigma-Aldrich, St. Louis, MO) at a dose of 44 mg/100 g body weight of FITC-labeled dextran. After four hours, mice were anesthetized with isoflurane, and 300–500 μl of blood was collected by cardiac puncture. Serum collected from blood samples by centrifugation for 15 minutes at 1500xg and 4°C was diluted with an equal volume of PBS. 100 μl of diluted serum was transferred to a 96-well plate, and FITC concentration was fluorometrically quantified by emission spectrometry (Promega, Madison, WI) at 528 nm using an excitation wavelength of 485 nm. Serum from mice not administered FITC-dextran was used to determine background. All concentrations were measured against a standard curve of serially diluted FITC-dextran.

### Bactericidal activity assay

The bactericidal activity assay was performed based on the procedure described by Udden et al.^83^.

Preparation of conditioned media from ileum tissue samples: 4–5 cm of the distal ileum was resected and immediately flushed with sterile-filtered ice-cold PBS to remove digesta. Ileum tissue samples were cut longitudinally, rinsed in sterile-filtered ice-cold PBS, and weighed. Tissue samples were disinfected in 5 ml DMEM/F12 media supplemented with 5% FBS and 1x antibiotics (penicillin, streptomycin, and vancomycin) for two hours at 37°C in a 95%O_2_/5%CO_2_ incubator. After disinfection, residual antibiotics were washed off by rinsing the samples three times with 5 ml antibiotic-free DMEM/F12 media supplemented with 5% FBS. Rinsed ileum tissue samples were then cut into 1 cm pieces using sterile scissors and transferred to 12-well cell culture plates containing fresh antibiotic-free DMEM/F12 media supplemented with 5% FBS. 1 ml of DMEM/F12 supplemented with 5% FBS was used per 100 mg of tissue. Samples were incubated at 37°C in an incubator with 5% CO_2_ and 95% O_2_ for 12 hours. Tissue supernatants (or conditioned media) were subsequently transferred into sterile 1.5 ml centrifuge tubes. Tissue debris was sedimented by centrifugation at 12,000 × g at 4°C for five minutes, and the conditioned media was used for the antibacterial activity assay.

Antibacterial activity assay: The prototypical Gram-negative bacteria, *Escherichia coli (E. coli strain HB101)*, was inoculated in 5 ml of Luria-Bertani (LB) broth and incubated overnight at 37°C with constant shaking at 250 rpm. Cultured bacteria were collected by centrifugation at 1,200 × g for 10 minutes at 4°C and then resuspended in fresh LB broth at a final concentration of 1×10^5^ cells/mL. 20 μL of the diluted bacteria were added to 500 μL of the ileum-derived conditioned media and incubated for one hour in an incubator maintained at 37°C and 95% O_2_/5% CO_2_. An additional 20 μL of the diluted bacteria were incubated with 500 μL of DMEM/F12 media supplemented with 5% FBS and 1x antibiotics (penicillin, streptomycin, and vancomycin; positive internal control) and with 500 μL antibiotic-free DMEM/F12 media containing 5% FBS (negative internal control). 500 μL of the ileum-derived conditioned media supplemented with 20 μL of fresh LB broth was also incubated along with the other samples to check for the presence of contamination (sham control). Thus, an experiment with each ileum-derived conditioned media constituted four groups: A) ileum supernatant + *E. coli*, B) ileum supernatant + LB broth, C) negative control, and D) positive control. A BFHI agar plate was divided into four quadrants, and 50 μL per group was evenly applied to each quadrant. The agar plate was incubated at 37°C, and bacterial colonies were counted 15–18 hours later. The experiment was repeated for ileum supernatants prepared from different mice.

The prototypical Gram-positive bacteria, *Lactobacillus reuteri* (*L.reuteri strain ATCC 53608)*, was cultured in de Man, Rogosa, and Sharpe (MRS) broth supplemented with 0.001% Tween 80 in a 95% O_2_/5% CO_2_ incubator maintained at 37°C. The cultured bacteria were collected by centrifugation, resuspended in fresh MRS broth, and serially diluted to a final concentration of 1 × 10^5^ cells/mL. The activity of the ileum tissue supernatants against *L. reuteri* was tested using the methodology described above for *E. coli*. Briefly, each ileum tissue supernatant experiment consisted of four groups: A) ileum supernatant + *L.reuteri*, B) ileum supernatant + MRS broth, C) DMEM/F12 media + 5% FBS + *L.reuteri* (negative control), and D) DMEM/F12 media + 5% FBS + 1x antibiotics (penicillin, streptomycin, and vancomycin) + *L.reuteri* (positive control). 50 μL per group was uniformly smeared onto MRS agar plates divided into four quadrants, and the total number of bacterial colonies formed was determined after incubation for 15–18 hours.

#### RNA isolation and qRT-PCR:

Total RNA was extracted from the ileum of placebo or morphine-pelleted mice orally administered with FMT on day 7 and from the ileum of mice injected repeatedly with saline or morphine and orally administered with 0.250 M sodium butyrate or its vehicle, saline, on day 5 using TRIzol reagent (ThermoFisher Scientific, Waltham, MA). RNA samples were treated with DNase 1 (RNase-free, ThermoFisher Scientific, Waltham, MA) to remove DNA contamination. Quantitative real-time polymerase chain reaction (qRT-PCR) was performed on a Mini-Opticon real-time PCR system (Bio-Rad, Hercules, CA) by using the iTaq Universal SYBR Green One-Step kit (Bio-Rad, Hercules, CA) as described previously^33^. Gapdh was used as the internal control. Primers used in this study were: murine Reg3γ forward, 5’-CGTGCCTATGGCTCCTATTGCT-3’; murine Reg3γ reverse, 5’-TTCAGCGCCACTGAGCACAGAC-3’; murine Gapdh forward 5’-CCATGGAGAAGGCTGGGG-3’; and murine Gapdh reverse 5’-CAAAGTTGTCATGGATGACC-3’ (Integrated DNA Technologies, Inc., Skokie, Illinois).

### Microbiome profiling

Fecal pellets were collected from saline-treated placebo-pelleted, butyrate-treated placebo-pelleted, saline-treated morphine-pelleted, and butyrate-treated morphine-pelleted mice. DNA was extracted using the QIAamp Fast DNA Stool Mini Kit according to the manufacturer’s protocols, and DNA was sent to CosmosID (Cosmosid Inc, Rockville, MD) and subjected to whole shotgun sequencing using the Illumina platform. An average of 5.921 M reads per sample was achieved with a minimum of 4.514M reads and a maximum of 8.816M reads. CosmosID’s k-mer based approach was used for taxonomic identification by comparing sequences to an in-house database. Profiles were analyzed using the filtered species-level data containing 297 bacterial species. Counts were renormalized to the mean number of reads with a pseudo count added to each bacterial species count and the counts were log10 transformed.

### Blinding

Experimenters were not blinded while performing experiments. However, separate investigators conducted the experiments to ensure reliability of results.

#### Data analysis:

Warm-water tail-withdrawal test and hot-plate test: Data represented as %MPE in Fig. 1 were evaluated by two-way ANOVA with FMT treatment and morphine treatment as the two independent variables. The Tukey’s multiple comparisons test was used for *post hoc* analysis. Data represented as %MPE in Fig. 3A were evaluated by two-way ANOVA with butyrate dose and morphine treatment as the two independent variables, and in Fig. 3B, by two-way ANOVA with butyrate treatment and morphine treatment as the two independent variables. Tukey’s *post hoc* test was used for multiple comparisons between groups.

Butyrate levels in blood, colon, and stool: Data in Fig. 2A were analyzed by two-way ANOVA with butyrate treatment and morphine treatment as the independent variables. Tukey’s multiple comparisons test was used for *post hoc* analysis. Data in Figs. 2B and 2C were analyzed by unpaired two-tailed Student’s t-test.

Intestinal permeability assay: Serum concentrations of FITC-dextran were evaluated in Fig. 4A by two-way ANOVA with FMT treatment and morphine treatment as the independent variables and in Fig. 4B by two-way ANOVA with butyrate treatment and morphine treatment as the independent variables. Tukey’s *post hoc* test was used for multiple comparisons between groups.

Bactericidal activity assay: The total number of bacterial colonies formed on agar plates were converted to colony forming units (CFU)/mL, such that CFU/mL = (number of colonies × dilution factor) / 50 μL. Data in Figs. 5A–C and 7A–D were evaluated by repeated-measures one-way ANOVA with the media as the independent variable. Tukey’s *post hoc* test was used for multiple comparisons between groups. CFU/mL data were transformed into percent bactericidal activity in Figs. 5D, 6E, and 8, such that % Bactericidal activity = {[(CFU/mL of antibiotic-free DMEM/F12 media + bacteria) - (CFU/mL of Ileum supernatant + bacteria)] / (CFU/mL of antibiotic-free DMEM/F12 media + bacteria)} * 100. Data in Fig. 5D was assessed by one-way ANOVA with treatment as the independent variable. The Holm-Sidak *post hoc* test was used for multiple comparisons between groups. Data in Fig. 7E was evaluated by two-way ANOVA with butyrate treatment and morphine treatment as the two independent variables. Multiple comparisons between groups were made using Tukey’s *post hoc* test. Data in Fig. 8 was evaluated by one-way ANOVA with treatment as the independent variable. Tukey’s multiple comparisons test was used for *post hoc* analysis.

qRT-PCR: Relative expression of Reg3γ to Gapdh was calculated using the 2^−ΔΔCt^ method, and values were expressed as fold change. Data were analyzed in Fig. 6A by two-way ANOVA with FMT treatment and morphine treatment as the independent variables and in Fig. 6B by two-way ANOVA with butyrate treatment and morphine treatment as the independent variables. Multiple comparisons between groups were made using Tukey’s *post hoc* test.

The threshold for statistical significance was P < 0.05. *Post hoc* analysis of the ANOVA was performed only for significant main effects or significant interactions. GraphPad Prism (version 9.4.1) was used for data analysis. Data are presented as mean ± SEM.

Microbiome analysis: The Permutational multivariate analysis of variance (PERMANOVA) using the Bray-Curtis dissimilarity index was utilized to evaluate the beta diversity of the fecal bacteria between the different groups. Results of the statistical analysis were obtained using the CosmosID Hub. The alpha diversity index, Chao1, for all the groups was determined using the CosmosID Hub. The data were analyzed by unpaired two-tailed Student’s t-test or two-way ANOVA, and Tukey’s *post hoc* test was used for pairwise comparisons using GraphPad Prism (version 9.4.1).

## Data Availability

The data supporting these findings have been deposited with NCBI BioProject at https://www.ncbi.nlm.nih.gov/bioproject (Reference PRJNA992486)
